# Oxidative Stress Markers and Sperm DNA Fragmentation in Men Recovered from COVID-19

**DOI:** 10.3390/ijms231710060

**Published:** 2022-09-02

**Authors:** Anastasiia D. Shcherbitskaia, Evgeniia M. Komarova, Yulia P. Milyutina, Mariia A. Ishchuk, Yanina M. Sagurova, Galina K. Safaryan, Elena A. Lesik, Alexander M. Gzgzyan, Olesya N. Bespalova, Igor Y. Kogan

**Affiliations:** D.O. Ott Research Institute of Obstetrics, Gynecology and Reproductive Medicine, 199034 St. Petersburg, Russia

**Keywords:** 8-hydroxy-2′-deoxyguanosine, nitrotyrosine, total antioxidant capacity, catalase, superoxide dismutase, uric acid, zinc, COVID-19, DNA fragmentation, sperm

## Abstract

SARS-CoV-2 negatively affects semen characteristics, impairs various biochemical processes in seminal fluid and within spermatogenic cells ultimately leading to male fertility decline. However, the distinct mechanisms, in particular, the role of oxidative stress on the consequences of coronavirus infection, have not been well investigated, which is the purpose of the present study. The standard semen parameters, its pro- and antioxidant system state, as well as the level of sperm DNA fragmentation, were assessed in 17 semen samples of men five months after the coronavirus infection and in 22 age-matched control patients. We determined that the DNA fragmentation rate negatively correlated with the period after coronavirus recovery, as well as seminal fluid superoxide dismutase activity and uric acid level. It was demonstrated that COVID-19 is not always associated with increased DNA fragmentation, allowing them to be considered as two independent factors. Thus, the most significant changes were noted in the samples of men after COVID-19 and abnormal TUNEL results: increased round cell number, decreased seminal fluid’s nitrotyrosine level, and total antioxidant capacity and Zn, as well as an increased 8-hydroxy-2′-deoxyguanosine level within spermatozoa. The data obtained indicate that increased DNA fragmentation and diminished semen quality in men can be the result of an imbalance in semen pro- and antioxidant components after COVID-19.

## 1. Introduction

The available data indicate that the human reproductive system is potentially vulnerable to the Coronavirus Disease 2019 (COVID-19). Thus, in adult men, a high expression of angiotensin-converting enzyme 2 (ACE2) was revealed in spermatogonia, spermatids, Leydig and Sertoli cells [1]. In this regard, it is assumed that severe acute respiratory syndrome coronavirus 2 (SARS-CoV-2) infection can contribute to testicular cell destruction and impaired spermatogenesis, ultimately leading to male infertility. At the same time, the data on the presence of a virus in human semen are still conflicting [2,3,4,5] and do not support the direct impact of SARS-CoV-2 on sperm cells functioning. Despite this, coronavirus infection was found to influence the hormonal profile responsible for maintaining male reproductive function [6]. Increased levels of pro-inflammatory cytokines [3,7] and leukocytes [3] in the seminal plasma of patients after COVID-19 were also reported to indicate a systemic inflammatory process even after recovery.

The recent meta-analysis reported on a negative impact of SARS-CoV-2 infection in relation to various semen characteristics, such as semen volume, sperm concentration, the total number of spermatozoa, sperm motility and progressive sperm motility [8,9]. At the same time, reports exist that the consequences of the COVID-19 can be serious, but reversible [10,11,12]. However, at this point there is no clear knowledge on duration necessary for sperm quality improvement. It was established that the total number, concentration and percentage of progressively motile spermatozoa were significantly lower in men during the first sampling (56 days after recovery), while sperm vitality and morphology did not change compared to controls [11]. Three weeks later, semen analysis revealed an improvement in the above-indicated parameters, while the percentage of morphologically abnormal spermatozoa decreased relative to the values obtained during the first sampling [11]. Based on the data obtained, the authors concluded that the consequences of the COVID-19 are observed during one spermatogenic cycle, which lasts about 74 days. However, another study reported on a significantly lower number of spermatozoa in patients with longer recovery period after COVID-19 (>90 days) compared to patients investigated in a period of less than three months after recovery [9]. Later, the same authors indicated semen quality restoration after about six months of having the disease [10].

The activation of caspases, DNA fragmentation, an increased number of reactive oxygen species (ROS) and nitric oxide generation are among the semen quality changes occurring at the molecular level due to coronavirus infection. Thus, an increased activity of caspase-3 and the elevated caspase-8 and -9 content were reported in the seminal plasma of patients after COVID-19 [7], all of which represent markers of cellular death. Additionally, the percent of TUNEL-positive sperm in COVID-19-infected patients was increased early in the disease and remained elevated over the next 60 days [7]. These data may indicate that COVID-19 causes significant cytological changes, DNA damage and sperm apoptosis. However, there is no possibility to judge the duration of these effects due to the limited number of studies.

Many researchers have investigated cellular mechanisms leading to DNA fragmentation over the past ten years due to the exceptional importance of maintaining the DNA integrity for human reproduction and healthy offspring. In addition to the rather well-known external DNA fragmentation inducers, free radicals, including ROS [13], acting both in testicles and in post-testicular regions [14,15] are believed to be one of the main causes of sperm DNA damage. Available studies demonstrate that COVID-19 causes a significant impairment of antioxidant system within men seminal plasma, as evidenced by a decreased activity of superoxide dismutase (SOD), as well as increased ROS 2 months after the recovery [7].

Despite the negative impact of SARS-CoV-2 on the sperm quality, there is still not enough data on the DNA fragmentation degree of semen and markers of oxidative stress after a coronavirus infection in the long term. Considering the above-listed facts, our study evaluated the parameters of semen, the level of DNA fragmentation, the markers of oxidative modifications of macromolecules (8-hydroxy-2′-deoxyguanosine, 8-OHdG; nitrotyrosine, NT) and antioxidant system components (total antioxidant capacity, TAC; the activity of catalase, CAT, and SOD; uric acid and Zn) in seminal plasma and spermatozoa of reproductive-aged men five (IQR: 4–7) months after coronavirus infection.

## 2. Results

### 2.1. Principal Components

**Figure 1:**
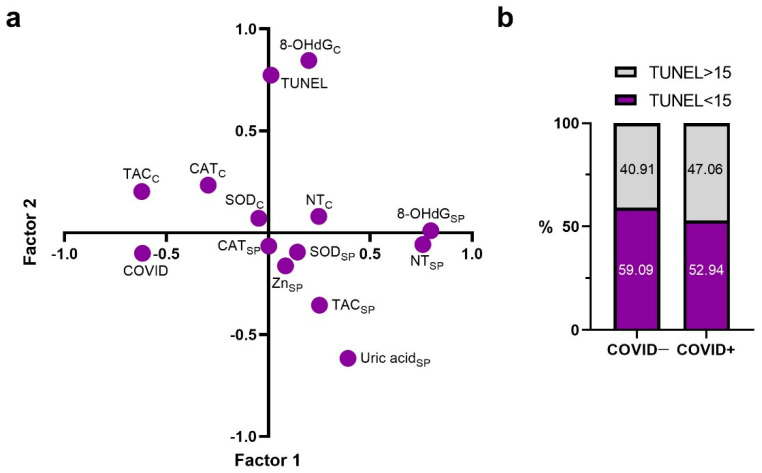
(**a**) A two-dimensional factor loading plot of the studied markers. 8-OHdG—8-hydroxy-2′-deoxyguanosine; CAT—catalase; NT—nitrotyrosine; SOD—superoxide dismutase; TAC—total antioxidant capacity; C (subscript)—cellular; SP (subscript)—in seminal plasma. (**b**) The plot of sample distribution with normal and abnormal TUNEL values in men after COVID-19 and without it.

In this study, in order to obtain general information about the connection between the investigated parameters and select the most significant one for its influence description, a factor analysis was carried out (Figure 1a). As a result, it was established that the first factor was more strongly associated with seminal plasma’s concentration of 8-OHdG in (a = 0.797), as well as the NT level (a = 0.758). In addition, this factor was associated to a much lesser degree with changes in such markers as TAC (a = −0.621) and COVID-19 positivity (a = −0.618). A change in the cellular 8-OHdG content was associated with the second factor (a = 0.845), also significantly affecting the changes in TUNEL (a = 0.773) and seminal plasma’s uric acid (a = −0.617). According to our results, cellular and seminal plasma’s SOD activity, as well as CAT activity, are controlled by other factors.

Due to the fact that COVID and TUNEL parameters are influenced by different non-depended factors, in our study we divided patients into four groups depending TUNEL rate and a history of coronavirus infection: “COVID−, TUNEL<15”,“COVID+, TUNEL<15”, “COVID−, TUNEL>15 ”,“COVID+, TUNEL>15 ”. Data on the number of samples with normal and pathological DNA fragmentation rate in men who have had the COVID-19 and without it are presented in Figure 1b.

### 2.2. Correlations with TUNEL and COVID Factors

Spearman rank correlation analysis established an association between the level of DNA damage and the number of months after the coronavirus infection (Table 1). So, it was demonstrated that the TUNEL level is reduced over the recovery time after the COVID-19. In addition, the level of DNA fragmentation in spermatozoa positively correlated with the rate of immotile spermatozoa and the number of round cells in the samples (*p* ≤ 0.05), the rate of spermatozoa with abnormal head morphology (*p* ≤ 0.01), the NT level and cellular CAT activity (*p* ≤ 0.05) in all samples investigated. TUNEL level was negatively associated with normal sperm morphology rate (*p* ≤ 0.001) and the rate of spermatozoa with mild degree of pathology (*p* ≤ 0.01). Reduced SOD activity and uric acid concentration in the seminal plasma (*p* ≤ 0.01) as well as increased sperm DNA damage were also established.

Spearman correlation revealed a negative association between the time after corona-virus infection and the percentage of sperm with head pathology (*p* ≤ 0.05). A positive correlation was also noted between the number of rehabilitation months after the COVID-19 and the percentage of sperm with a mild degree of pathology (*p* ≤ 0.05), sperm with tail pathology (*p* ≤ 0.01), as well seminal plasma TAC (*p* ≤ 0.001).

### 2.3. Semen Quality Parameters

The comparative analysis confirmed no difference in semen parameters such as the rate of spermatozoa with progressive motility, non-progressive motility and immotile cells in men after COVID-19, in groups with normal and abnormal DNA fragmentation rate when compared to the results of patients without a history of SARS-CoV-2 (Table 2).

Along with the standard assessment, the sperm morphological investigation also revealed no differences in the rate of spermatozoa with normal morphology, with head pathology, midpiece and tail pathologies between the “COVID−, TUNEL<15” and “COVID+, TUNEL<15” groups, as well as “COVID−, TUNEL>15”and “COVID+, TUNEL>15” groups.

However, we discovered a reliably increased (*p* ≤ 0.05) round cell number in samples of “COVID+, TUNEL>15” group in comparison to “COVID−, TUNEL>15” group.

### 2.4. Antioxidant System Components

**Figure 2:**
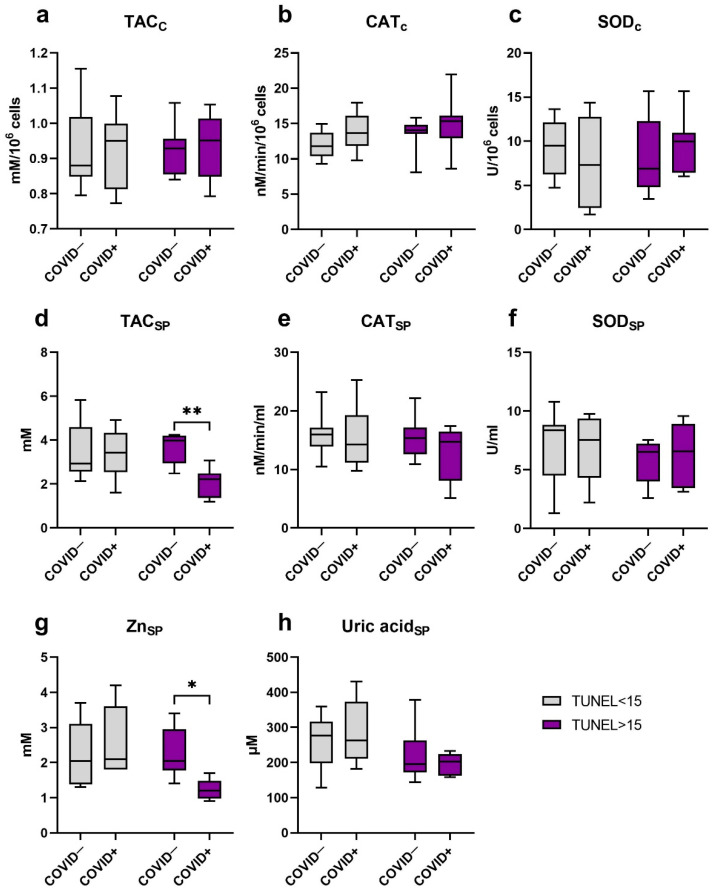
The antioxidant components level in sperm and seminal plasma of men after COVID-19, depending on DNA fragmentation level. (**a**) Total antioxidant capacity in cells. (**b**) Catalase activity in cells. (**c**) Superoxide dismutase activity in cells. (**d**) Total antioxidant capacity of seminal plasma. (**e**) Catalase activity in seminal plasma. (**f**) Superoxide dismutase activity in seminal plasma. (**g**) Zn level in seminal plasma. (**h**) Uric acid concentration in seminal plasma. C (subscript)—cellular; SP (subscript)—in seminal plasma. Values are expressed as Me [25, 75%], whiskers—min-max. Significance of difference between the «COVID−, TUNEL<15» and «COVID+, TUNEL<15», as well as «COVID−, TUNEL>15» and «COVID+, TUNEL>15» groups were determined by the Mann–Whitney U test. *—*p* ≤ 0.05; **—*p* ≤ 0.01.

The analysis of antioxidant system markers in ejaculates’ cellular fraction such as TAC, SOD and CAT activities revealed no reliable differences between the groups investigated (Figure 2a–c).

At the same time, no differences in uric acid (Figure 2h), SOD (Figure 2f) and CAT (Figure 2e) activity were noted in the seminal plasma of “COVID−, TUNEL<15” and “COVID+, TUNEL<15” groups, as well as “COVID−, TUNEL>15” and “COVID+, TUNEL>15” groups.

The levels of Zn and TAC in the seminal plasma of men after COVID−19 and normal DNA fragmentation rate did not differ from the same values in the “COVID−, TUNEL<15” group (Figure 2d,g).

The changes in seminal plasma Zn level and TAC were found when compared in the “COVID−, TUNEL>15” and “COVID+, TUNEL>15” groups. Thus, a reliable decrease (*p* ≤ 0.05) of Zn content in seminal plasma of patients with abnormal DNA fragmentation and the COVID-19 infection about five months before the sampling was observed. In addition, those patients had seminal plasma TAC reduction (*p* ≤ 0.01).

### 2.5. Oxidative Modification of DNA and Proteins

**Figure 3:**
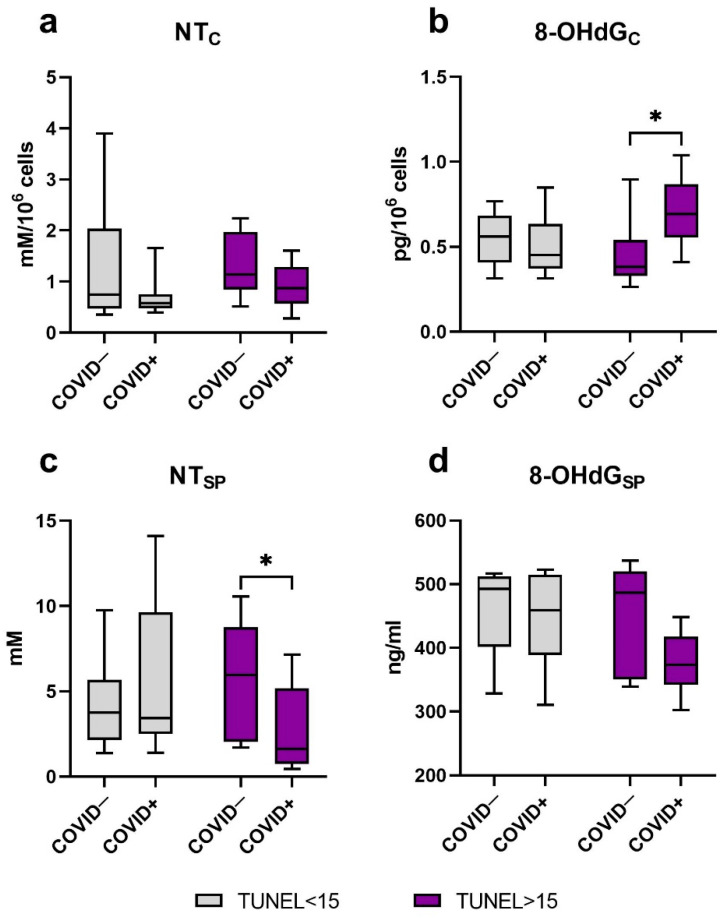
The level of macromolecule oxidative modification in seminal plasma and spermatozoa of men after COVID-19, depending on DNA fragmentation level. (**a**) Nitrotyrosine content in cells. (**b**) 8-hydroxy-2′-deoxyguanosine level in cellular fraction. (**c**) Nitrotyrosine concentration in seminal plasma. (**d**) 8-hydroxy-2′-deoxyguanosine level in seminal plasma. C (subscript)—cellular; SP (subscript)—in seminal plasma. Values are expressed as Me [25, 75%], whiskers—min-max. Significance of difference between the «COVID−, TUNEL<15» and «COVID+, TUNEL<15», as well as «COVID−, TUNEL>15» and «COVID+, TUNEL>15» groups were determined by the Mann–Whitney U test. *—*p* ≤ 0.05.

The recent study reported no reliable differences in such markers of oxidative damage as NT in spermatozoa (Figure 3a) and 8-OHdG in seminal plasma (Figure 3d) of patients from “COVID−, TUNEL<15” and “COVID+, TUNEL<15” groups as well as “COVID−, TUNEL>15” and “COVID+, TUNEL>15” groups.

However, we noted an increase in ejaculates’ cellular fraction 8-OHdG in men after a coronavirus infection with the TUNEL level >15 compared to the “COVID−, TUNEL>15” group (*p* ≤ 0.05), while this marker did not differ in men with normal DNA fragmentation values (Figure 3b).

During the analysis of seminal plasma NT concentration, no reliable differences were established between the “COVID−, TUNEL<15” and “COVID+, TUNEL<15” groups. At the same time, seminal plasma NT level was higher in men from “COVID−, TUNEL>15” group (*p* ≤ 0.05) when compared to “COVID+, TUNEL>15” group (Figure 3c).

### 2.6. Correlations between the Studied Variables of Oxidative Stress

**Figure 4:**
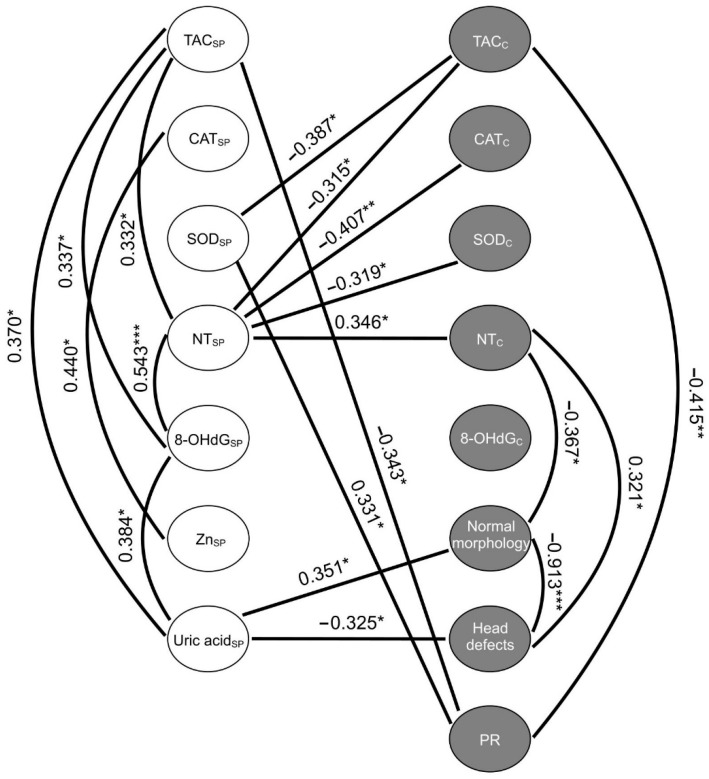
The correlation pleiades of the antioxidant components, markers of oxidative modification of proteins and DNA in seminal plasma and sperm, as well as some semen analysis characteristics in the general patient sample. PR—progressive motility; 8-OHdG—8-hydroxy-2′-deoxyguanosine; CAT—catalase activity; NT—nitrotyrosine; SOD—superoxide dismutase activity; TAC—total antioxidant capacity; C (subscript)—cellular; SP (subscript)—in seminal plasma. The Spearman correlation coefficient was used to identify the strength and direction of relationship between studied markers. *—*p* ≤ 0.05; **—*p* ≤ 0.01; ***—*p* ≤ 0.001.

Spearman’s rank correlation between the markers of oxidative stress and antioxidant components in seminal plasma and spermatozoa, as well as the rate of morphologically normal forms, the forms with head defects and spermatozoa with progressive motility in the general sample are presented in Figure 4 in the form of correlation pleiades.

The strongest negative association was observed between the rate of morphologically normal sperm and the rate of sperm with head defects (*p* ≤ 0.001). At the same time, the cellular NT level negatively correlated with the rate of normal forms (*p* ≤ 0.05) and positively correlated with the rate of sperm with head pathology (*p* ≤ 0.05). A similar trend was established for seminal plasma uric acid in relation to the above-listed parameters (*p* ≤ 0.05). Elevation of seminal plasma 8-OHdG along with increasing uric acid concentration (*p* ≤ 0.05) and NT level was observed (*p* ≤ 0.001). Seminal plasma NT was negatively correlated with such markers within spermatozoa as TAC (*p* ≤ 0.05), SOD activity (*p* ≤ 0.05) and CAT activity (*p* ≤ 0.01). It was noted that increasing NT level within spermatozoa was associated with increasing levels of the mentioned marker in seminal plasma (*p* ≤ 0.05). The cellular TAC was negatively associated with SOD activity in seminal plasma (*p* ≤ 0.05) and the rate of sperm with progressive motility (*p* ≤ 0.01), while the decline in the rate of the latter in ejaculate was accompanied by lower seminal plasma SOD (*p* ≤ 0.05).

In addition, along with the measurement of the investigated parameters in general patient samples, we also evaluated the association of oxidative stress and antioxidant system markers separately in each group in order to assess the changes between markers depending on various factors.

**Figure 5:**
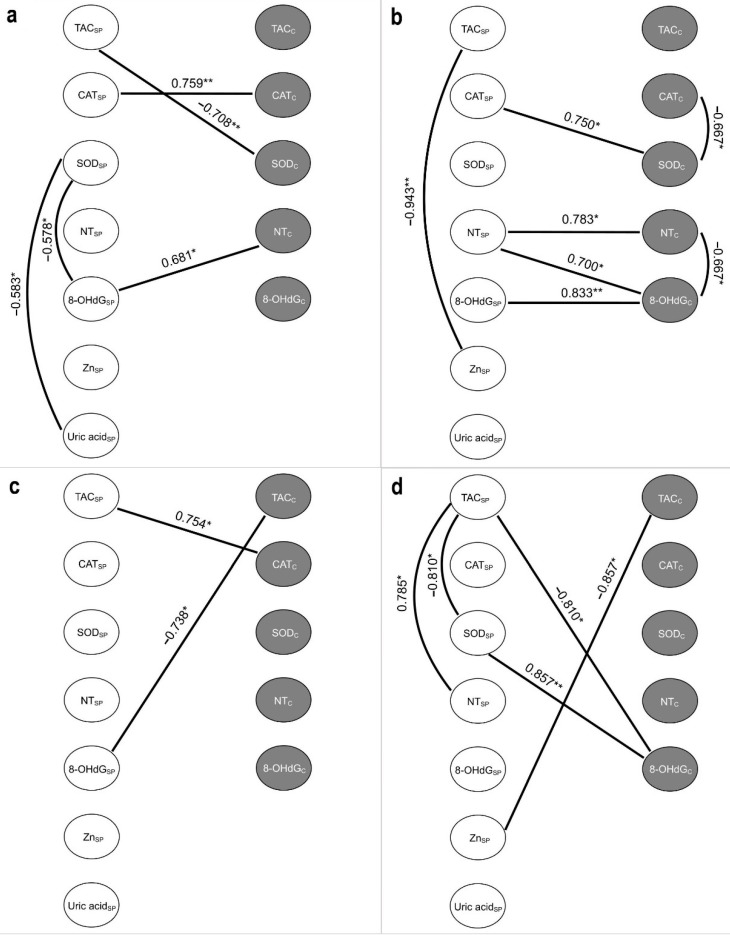
Correlation pleiades of the studied markers in groups (**a**) COVID−, TUNEL<15; (**b**) COVID+, TUNEL<15; (**c**) COVID−, TUNEL>15; (**d**) COVID+, TUNEL>15. 8-OHdG—8-hydroxy-2′-deoxyguanosine; CAT—catalase; NT—nitrotyrosine; SOD—superoxide dismutase; TAC—total antioxidant capacity; C (subscript)—cellular; SP (subscript)—in seminal plasma. The Spearman correlation coefficient was used to identify the strength and direction of the relationship between studied markers. *—*p* ≤ 0.05; **—*p* ≤ 0.01.

Figure 5a contains the results of Spearman’s analysis for “COVID−, TUNEL<15” group. The strongest positive relationship was detected between CAT activity in seminal plasma and spermatozoa (*p* ≤ 0.01). The increasing seminal plasma TAC was associated with a decreasing cellular SOD activity (*p* ≤ 0.01), while the latter was negatively related to seminal plasma 8-OHdG and uric acid (*p* ≤ 0.05) and positively to the NT level in spermatozoa (*p* ≤ 0.05).

Despite the normal TUNEL values, the determined associations changed after COVID-19 infection (Figure 5b). Seminal plasma 8-OHdG elevation was associated with its increase in spermatozoa (*p* ≤ 0.01). The seminal plasma NT level was positively correlated with NT and 8-OHdG in cellular fraction (*p* ≤ 0.05), while the latter was inversely related to each other (*p* ≤ 0.05). A decline in SOD activity within spermatozoa was accompanied by increased cellular CAT activity (*p* ≤ 0.05), but decreased seminal plasma CAT activity (*p* ≤ 0.05). A strong negative association was also demonstrated between Zn level and TAC in seminal plasma (*p* ≤ 0.01).

Only two significantly reliable associations were noted for the markers studied in the “COVID−, TUNEL>15” group (Figure 5c). Seminal plasma TAC increased with elevating cellular CAT activity (*p* ≤ 0.05), while negative correlation was revealed between spermatozoa TAC and seminal 8-OHdG (*p* ≤ 0.05).

In the seminal plasma of “COVID+, TUNEL>15” group (Figure 5d), an increased TAC was associated with increasing NT content (*p* ≤ 0.05) and decreasing SOD activity (*p* ≤ 0.05). The activity of the latter was positively related to 8-OHdG level within spermatozoa (*p* ≤ 0.01), which in turn was negatively associated with seminal plasma TAC (*p* ≤ 0.05). The Zn concentration in seminal plasma also decreased with increasing TAC in spermatozoa (*p* ≤ 0.05). In addition, we established a negative relationship between the volume of semen and the number of months after coronavirus infection in this group of patients (r_s_ = −0.736, *p* ≤ 0.05).

## 3. Discussion

According to the meta-analysis [8] the existing research results on the COVID-19 effect on male reproductive health should be interpreted with caution. For example, ignoring some factors that, along with a viral infection, can potentially affect the quality of semen and sperm and, consequently, fertility, is a significant drawback of previous studies. The studies also did not report the socio-demographic factors, such as drug abuse, tobacco and alcohol consumption, all of which represent risk factors for reducing sperm quality. In addition, despite the fact that the age of the patients is an important factor in risk assessment, the information about the age of patients was often absent in the studies of COVID-19 effect on male reproductive health. The present study attempted to take into account the existing limitations of previous research projects by strict exclusion criteria. To our knowledge, the present study for the first time considers COVID-19 and TUNEL as two independent factors in the analysis of semen parameters and the pro- and antioxidant markers in the cells and male seminal plasma. So, as a result of the study, the most significant changes were noted in the “COVID+, TUNEL>15” group, most likely due to the combination of the two independent affecting factors studied. At the same time, the absence of differences in groups with normal TUNEL level may indicate a faster restoration of the pro- and antioxidant system balance in such men, or the absence of the COVID-19 effect on the parameters analyzed.

Despite the fact that the evaluation of the standard semen parameters and morphological features of spermatozoa in patients did not reveal significant differences, the “COVID+, TUNEL>15” group exerted an increased number of round cells in semen, including immature germ cells and leucocytes, which are known to be the main sources of ROS in the ejaculate [16]. In addition, our finding of a semen volume decline over the time after the coronavirus infection in the “COVID+, TUNEL>15” group is partially consistent with other studies [9].

An increased number of TUNEL-positive apoptotic cells in the testicles of COVID-19 deceased men was established, caused in some views by the oxidative stress due to an elevated circulating ROS and suppressed activity of glutathione peroxidase [17]. The evaluation of spermatozoa revealed that coronavirus infection promotes fragmentation of their DNA and, as a result, cell death via both extrinsic and intrinsic pathways of apoptosis [7]. In a study comparing the sperm DNA fragmentation rates in semen samples obtained from patients with the recovery period <90 and >90 days, no temporary differences were noted [9]. However, our study revealed a negative association between the TUNEL assay results and the sample collection time after COVID-19, indicating that sperm DNA fragmentation level decreases over the time after recovery.

In addition, Maleki and Tartibian reported on lower percent of sperm having normal morphology in the setting of increased ROS in seminal plasma [7]. Our study also revealed an inverse correlation between such markers of oxidative stress as NT and the percent of morphologically normal sperm. Spermatozoa are known to generate nitric oxide and superoxide radical in small amounts, irreversibly reacting with each other and resulting in peroxinitrite formation which, in turn, represents a powerful oxidant and/or nitrating agent, attacking sperm DNA, proteins and lipids [18]. Besides, peroxinitrite induces nitration of tyrosine residues, resulting in NT production and spermatozoa damage or degeneration [18]. The relationship between NT and sperm motility decline was established [19]. Moreover, increased seminal plasma’s NT was reported in men with idiopathic causes of infertility [20]. Despite the discovered association between semen NT level and standard parameters of semen analysis in other pathologies, such studies in relation to patients after COVID-19, as far as we know, have not yet been conducted. Surprisingly, contrary results on lower seminal plasma’s NT content in “COVID+, TUNEL>15” patients were obtained. However, similar data on decreased seminal plasma NT content in men with various forms of pathozoospermia were reported in another study [21]. According to the authors, the changes discovered may serve as important marker of the spermatozoa quality, due to the fact that reactive forms are also necessary for the normal functioning of these cells. In addition, Kalezic et al. reported on the negative relation between NT and sperm qualities in oligoastenoteratospermia [21]. Summarizing the results of previous studies and our data, we conclude that such markers as seminal plasma NT are also of a high importance for functional spermatozoa condition analysis. It is worth noting that the analysis of the entire patient population in our study revealed this particular parameter to be most often significantly associated with different markers of pro- and antioxidant systems in seminal plasma (8-OHdG and TAC) and spermatozoa (NT, CAT, SOD and TAC). At the same time, when considering correlation within individual groups of patients after COVID-19, the NT level remains associated with other parameters despite a lower number of people in the sample, emphasizing once again the importance of monitoring this marker after coronavirus infection.

A reliable oxidative damage of sperm DNA have been found in “COVID+, TUNEL>15” patients. According to these unique data, even mild COVID-19 infection can cause persistent long-term effects that could possibly affect the quality of the offspring. Despite the absence of reliable correlation between the degree of sperm DNA fragmentation and the level of oxidative DNA damage in the lysate, a positive association between the level of 8-OHdG and DNA damage of spermatozoa was reported in other studies evaluating the influence of various pathologies on male reproductive health [22,23,24,25,26]. Our results, however, are in line with other studies, confirming that simultaneous oxidative DNA damage with a high fragmentation rate was present only in a small number of spermatozoa and to a greater extent in cells with increased 8-OHdG [27].

Coronavirus infection was reported to cause an alteration in the semen antioxidant properties [7,17,28]. Many studies determined the TAC while evaluating semen antioxidant properties [28,29,30,31]. We confirmed an increase in the seminal plasma TAC in patients over the time after a coronavirus infection, which is consistent with other studies [28]. However, such a general assessment of antioxidant protection in biological sample does not allow us to conduct an accurate qualitative and quantitative analysis of particular antioxidants. For instance, no significant changes in SOD and CAT activity in seminal plasma and spermatozoa of men after COVID-19 from groups both with normal and elevated DNA fragmentation rate were noted. The negative association established in this particular study between the degree of DNA fragmentation and SOD activity in seminal plasma is consistent with the data of other studies on patients after COVID-19 [7]. Interestingly, the seminal plasma SOD activity was positively associated with the rate of sperm with progressive motility [7], also confirmed by the results of our study. In contrast to the general trend, one meta-analysis [32] did not confirm significant changes in seminal plasma SOD activity in patients with various forms of infertility, suggesting that not all antioxidant system components make the same contribution to maintaining sperm normal functioning. Thus, one of the possible explanations for seminal plasma TAC decline in “COVID+, TUNEL>15” patients, is perhaps, at least partially, in the interference of non-enzymatic antioxidant protection components such as vitamins and minerals.

Zn deficiency was found to be associated with arrest of spermatogenesis at the stages of round and elongated spermatids [33]. Reduced seminal plasma Zn levels were reported in infertile men [34,35]. Furthermore, high intracellular Zn concentration was established to inhibit RNA-virus replication, including SARS-CoV-2 [36]. In our study, lower seminal plasma Zn level in patients after COVID-19 and values of TUNEL>15 was found. Considering the antioxidant properties of Zn, its deficiency in seminal plasma can lead to oxidative damage of macromolecules caused by ROS. Besides, the analysis of samples with globozoospermia revealed an association between diminished Zn levels and high DNA fragmentation rate, which, according to the authors, may indicate not only an oxidative stress development but also the effect of Zn in maintaining the integrity of sperm DNA [37]. In our study, despite the absence of association between Zn level and TUNEL rate, the “COVID+, TUNEL>15” group exerted a decrease in seminal plasma Zn level along with elevated 8-OHdG in the cell fraction.

It was demonstrated that only urates along with ascorbic acid and tyrosine comprise 37% of the semen antioxidant activity [38]. The uric acid was discovered to be the most effective scavenger for peroxinitrite within spermatozoa [39], resulting in its positive effect on cell functioning and fertilization potential. Uric acid is the final product of purine nucleotides (guanosine and adenosine) catabolism in humans and higher primates [40]. This possibly explains the positive association we discovered between uric acid level and 8-OHdG seminal plasma content in the general patient population. Our data are also consistent with the results of other studies, which found no reliable association between cellular 8-OHdG and seminal plasma uric acid concentrations in men [41].

Since the oxidative stress is considered by many authors to be the main cause of DNA fragmentation, antioxidant supplementation for ROS level reduction in seminal plasma is commonly practiced for sperm parameters and fertility improvement [42]. In view of the fact that in the group of men recovered after coronavirus infection and with increased DNA fragmentation, an imbalance of the pro- and antioxidant systems components was noted, the strategy of antioxidant therapy can probably be applied in the management of such patients. Vitamin E, folic acid, Zn, vitamin C, L-carnitine and selenium were reported to be the most well studied and commonly used antioxidant agents for the male infertility treatment [43,44,45,46,47,48,49,50,51,52]. In addition, the influence of natural antioxidants in DNA fragmentation reduction, such as, for instance, ellagic acid, is actively being studied [53]. Our results suggest that high level of DNA fragmentation may not be accompanied by oxidized macromolecules level elevation and additional factors such as, for example, coronavirus infection, can be required to alter the pro- and antioxidant systems balance in semen. Therefore, the absence of oxidative stress verification in men and monitoring of the ROS level during therapy is a potential problem [54], since there is a risk of reductive stress induction due to excessive therapy with antioxidants [55].

This study has some limitations, including a relatively small sample size and a long period of sample collection. We did not specify the virus strains in affected patients, which could probably influence the results. Despite of the samples collection length, all the recruited patients had a verified diagnosis of COVID infection (PCR). During the infection, the symptoms were mild. None of them was admitted to the hospital and the treatment included only supportive measures (fluid intake, fever relief). In addition, we did not perform the semen analysis during the COVID-19 infection itself and did not perform the detection of the viral particles within the semen. Despite this, we tried to overcome the named limitations with strict inclusion criteria into the study, as well as with application of adequate statistical analysis methods. Future investigations with a larger number of patients, both in the general sample and within the groups, are warranted. Additionally, in order to assess the duration of semen pro- and antioxidant systems imbalance, it is worth analyzing the above-mentioned in this study markers at other time intervals, both up to and after five months of coronavirus infection.

## 4. Materials and Methods

### 4.1. Patients

The study was approved by the ethics committee of the D.O. Ott Institute of Obstetrics, Gynecology, and Reproductive medicine (protocol code 9 as of 4 September 2020). All participants gave written informed consent and completed a questionnaire which included information regarding age, body weight, height, general health, lifestyle, consumption of alcohol and drugs and smoking.

Semen samples were obtained from COVID-19 recovered patients (n = 17) attending the Department of Reproductive Medicine in the D.O. Ott Institute of Obstetrics, Gynecology, and Reproductive medicine between 16 October 2020, and 04 April 2022. The median period of recovery was five (IQR: 4–7) months. The control group was composed of 22 men of matching ages, who had not suffered from COVID-19. Exclusion criteria were antioxidant and vitamin supplementation, use of drugs such as habitual drugs, occupational and environmental exposures to potential reproductive toxins, BMI > 25. Patients were also excluded from analysis if they had azoospermia, varicocele, cryptorchidism, prostatitis, urinary tract infection, genital trauma, testicular torsion, inguinal or genital surgery, sexually transmitted disease, chronic illness and serious systemic diseases.

### 4.2. Semen Analysis and Preparation

Semen samples were collected from men after 3–5 days after abstinence by masturbation. After liquefaction for 15–60 min, semen samples were evaluated for sperm motility, concentration, and morphology, in accordance with the 2010 guidelines of the World Health Organization (WHO). The characteristics of patients and their semen quality are presented in Table 3.

After semen analysis, each fresh semen sample was divided into two aliquots: one immediately served for TUNEL staining and the other centrifuged at 3000× *g*, +4 °C for 15 min to obtain seminal plasma and a sperm pellet. The sperm cell pellet was washed three times in the phosphate buffer saline (PBS, pH 7.4, Sigma-Aldrich, St. Louis, MO, USA) and centrifuged for 15 min at 3000× *g*, +4 °C. After that 20 million sperm cells were homogenized in RIPA lysis buffer (Sigma-Aldrich, St. Louis, MO, USA), centrifuged for 15 min at 10,000× *g*, +4 °C. Seminal plasma and supernatant from the sperm cell pellet were frozen at −80 °C until oxidative stress parameters analysis.

### 4.3. Sperm DNA Fragmentation

The evaluation of sperm DNA fragmentation was performed by terminal deoxynucleotidyl transferase-mediated dUTP nick-end labeling assay (TUNEL) or an in situ Cell Death Detection Kit, using fluorescein (Roche Diagnostics, Germany) according to the manufacturer instructions. Semen samples were applied to a slide plate, dried, fixed for 24 h at a room temperature and rinsed using PBS. The samples were then fixed by immersion into 4% formaldehyde solution for 10 min. 0.1% Triton X-100 (Sigma-Aldrich, St. Louis, MO, USA) in sodium citrate 0.1% (Sigma-Aldrich, St. Louis, MO, USA) was added to the samples for 15 min at −20 °C and rinsed twice with PBS. The samples were mixed with the kit TUNEL assay reaction at 37 °C for 60 min in a humid chamber and rinsed twice with PBS. The results obtained were investigated using fluorescence microscopy under magnification ×1000. Bright green fluorescence within spermatozoa (wavelength—488 nm) characterized damaged (fragmented) DNA, while blue (DAPI, H-1200, Vectashield Vector Labs, Newark, CA, USA) fluorescence was indicative of normal DNA structure. The number of the cells counted was equal to 2000. The percentage of sperm with DNA fragmentation from the total number of sperm analyzed was calculated. The cut-off value of 15 for TUNEL was determined at our laboratory on the basis of the evaluation of 20 healthy sperm donors after proven clinical pregnancy.

### 4.4. Assessment of Antioxidant Markers

#### 4.4.1. Total Antioxidant Capacity

Trolox equivalent antioxidant capacity methodology was used to evaluate the seminal plasma and spermatozoa TAC. Total antioxidant capacity was determined spectrophotometrically, using an Antioxidant assay kit (709001, Cayman Chemical Co., Ann Arbor, MI, USA). Before analysis, seminal plasma and sperm supernatant were diluted by adding Assay Buffer. Antioxidants inhibit the oxidation of 2,2′-azino-bis(3-ethylbenthiazoline)-6-sulphonic acid by the ferryl myoglobin-H_2_O_2_ system. TAC was measured by determining the absorbance at 405 nm using plate reader (EL 800, BioTek Instruments, Winooski, VT, USA). Antioxidant concentration was expressed as millimolar trolox equivalents (mmol/L for seminal plasma and mmol/10^6^ cells for spermatozoa). Standards and samples were measured in triplicate.

#### 4.4.2. Superoxide Dismutase Activity

The activity of SOD in seminal plasma and sperm cells was analyzed using a colorimetric Superoxide Dismutase Assay Kit (706002, Cayman Chemical Co., Ann Arbor, MI, USA) according to the recommendations of the manufacturer. Briefly, 10 μL of the seminal plasma or cell lysate was added in the well of 96-well plate containing 200 μL Radical Detector. The reaction was initiated by adding 20 μL of Xanthine Oxidase. The plate was incubated on a shaker (Elmi Sky LineV-3, Riga, Latvia) for 30 min at room temperature. SOD activity was measured by the degree of inhibition of formazan dye formation following the reaction of superoxide radicals with tetrazolium salt that was detected at 450 nm and expressed as U/mL seminal plasma or U/10^6^ cells. Standards and samples were measured in triplicate.

#### 4.4.3. Catalase Activity

The seminal and cellular CAT activity was measured using Catalase Assay Kit (707002, Cayman Chemical Co., Ann Arbor, MI, USA) according to the recommendations of the manufacturer’s protocol. Briefly, 20 μL of the seminal plasma or cell lysate was added to the well of 96-well plate containing 100 μL Assay Buffer and 30 μL methanol. The reaction was initiated by adding 20 μL of Hydrogen Peroxide. The plate was incubated on a shaker for 20 min at room temperature. 30 μL of Potassium Hydroxide and 30 μL of Catalase Purpald (Chromogen) were added to each well to terminate the reaction. After adding 10 μL of Catalase Potassium Periodate, the enzyme activity was measured by determining the absorbance at 540 nm using plate reader CLARIOstar Plus (BMG Labtech, Ortenberg, Germany). CAT activity was expressed as nmol of formaldehyde produced/min/mL of seminal plasma or nmol of formaldehyde produced/min/10^6^ cells. Standards and samples were measured in triplicate.

### 4.5. Assessment of Oxidative Modifications of Macromolecules

#### 4.5.1. Nitrotyrosine Content

Nitrated seminal plasma and cellular proteins were assayed by sandwich enzyme-linked immunosorbent assay using Nitrotyrosine ELISA Kit (HK501, Hycult Biotech, Uden, Netherlands) and following the instructions provided by the manufacturer. The amount of nitrotyrosine was measured at 450 nm. Standards and samples were measured in triplicate.

#### 4.5.2. Oxidative DNA Damage

8-OHdG, an adduct of the oxidized guanine base, was detected to evaluate oxidative damage of DNA. The levels of 8-OHdG in seminal plasma and cells were measured using the DNA/RNA Oxidative Damage (High Sensitivity) ELISA Kit (589320, Cayman Chemical Co., Ann Arbor, MI, USA). The absorbance of each sample was determined in a plate reader at a wavelength of 405 nm and the levels of 8-OHdG were calculated from a standard curve. Samples were measured in triplicate.

### 4.6. Zinc Analysis in Seminal Plasma

Zn concentration in seminal plasma was analyzed using a diagnostic reagent kit (DIALAB, Wiener Neudorf, Austria). Zn forms a red chelate complex with 2-(5-Bromo-2-pyridylazo)-5-(N-propyl-N-sulfo-propylamino)-phenol. Absorbance was measured at 560 nm using automatic immunochemical analyzer UniCel DxI 600 (Beckman Coulter Life Science, Indianapolis, IN, USA).

### 4.7. Uric acid Analysis in Seminal Plasma

Uric acid level in seminal plasma was analyzed using a Uric Acid Reagent Kit (Beckman Coulter Life Science, Indianapolis, IN, USA). Uric acid in the sample was oxidized by uricase to form allantoin and hydrogen peroxide. Hydrogen peroxide reacts with 4-aminoantipyrine and 3,5-dichloro-2-hydroxyienzene sulfonate in a peroxidase catalyzed reaction to form a colored product. The absorbance was measured at 520 nm using automatic immunochemical analyzer UniCel DxI 600 (Beckman Coulter Life Science, Indianapolis, IN, USA).

### 4.8. Statistical Analysis

The normality of the data was tested using the Shapiro–Wilk normality test. Variance was analyzed by the Mann–Whitney U-test and Kruskal–Wallis H-test. Data were reported as box-and-whisker plots displaying medians (25th, 75th percentile) and the maximum and minimum as “whiskers”. The Spearman correlation coefficient (r_s_) was used to identify the strength and direction of relationship between studied markers. When comparing the markers measured in the nominal scale, Pearson’s Chi-square index (χ^2^) was used. For a comprehensive and compact description of the objects of study, factorial analysis by the principal components was applied; the method used was the Varimax rotation method with Kaiser normalization. The calculation of factor loadings (a) was carried out, which are interpreted as correlations between the corresponding studied parameters and individual factors (hypothetical, not directly measured, hidden features, to some extent related to the measured markers). The optimal number of factors was determined by using the scree plot. Statistical analysis was performed using the STATISTICA 10.0 software. Values of *p* ≤ 0.05 were considered statistically significant.

## 5. Conclusions

The present study demonstrates that the sperm DNA fragmentation rate negatively correlates with the recovery time after the coronavirus infection. However, despite the detected correlation, the analysis of the main components revealed that DNA fragmentation and COVID-19 are influenced by two independent factors, dividing patients included into four distinct groups. The most significant changes were noted in the group of men after COVID-19 and with an abnormal TUNEL rate (reduced TAC, Zn and NT levels in seminal plasma, as well as an increased DNA oxidative damage). In patients after COVID-19, the seminal plasma TAC increased over time after rehabilitation. However, this marker reflects the whole antioxidant status and does not give an idea of changes in its individual components. At the same time, the seminal plasma NT, which is reliably correlated to the markers investigated, can be probably considered one of indicators in the functional state of spermatozoa. It is also worth paying attention to the fact that the level of 8-OHdG did not correlate with TUNEL value, probably indicating that the occurrence of 8-OHdG, as a hallmark of oxidative stress, scarcely mirrored the presence of DNA fragmentation and vice versa.

## Figures and Tables

**Table 1:** Matrix of correlation coefficients of the investigated parameters with TUNEL and COVID factors.

Parameters	R_s_	
	TUNEL	Months after COVID-19
**Months after COVID−19**	−0.500 *	1.000
**Age (years)**	−0.033	−0.064
**BMI, kg/m^2^**	−0.037	0.089
**PR**	−0.281	−0.269
**NP**	−0.288	0.338
**IM**	0.398 *	0.184
**Round cells**	0.343 *	−0.239
**Normal morphology**	−0.559 ***	0.447
**Mild degree of pathology**	−0.443 **	0.523 *
**Head defects**	0.481 **	−0.534 *
**Midpiece defects**	−0.244	0.199
**Tail defects**	−0.136	0.674 **
**TAC_C_**	0.232	0.231
**TAC_SP_**	−0.095	0.751 ***
**NT_C_**	0.340 *	−0.012
**NT_SP_**	−0.114	0.401
**CAT_C_**	0.385 *	−0.288
**CAT_SP_**	0.023	−0.004
**SOD_C_**	0.070	−0.402
**SOD_SP_**	−0.442 **	−0.354
**8−OHdG_C_**	0.179	−0.391
**8OHdG_SP_**	−0.153	0.031
**Uric acid_SP_**	−0.414 **	0.216
**Zn_SP_**	−0.279	0.366

*—*p* ≤ 0.05; **—*p* ≤ 0.01; ***—*p* ≤ 0.001. BMI—body mass index; PR—progressive motility; NP—nonprogressive motility; IM—immotility; 8-OHdG—8-hydroxy-2′-deoxyguanosine; CAT—catalase; NT—nitrotyrosine; SOD—superoxide dismutase; TAC—total antioxidant capacity; C (subscript)—cellular; SP (subscript)—in seminal plasma.

**Table 2:** Median and interquartile range of standard semen analysis parameters, selected morphological defects in the groups of men.

Parameters	COVID−, TUNEL<15	COVID+, TUNEL<15	COVID−, TUNEL>15	COVID+, TUNEL>15
**Progressive motility (PR, %)**	60.00 [49.00–70.00]	62.00 [56.00–67.00]	53.00 [41.00–63.00]	62.00 [49.00–68.00]
**Nonprogressive motility (NP, %)**	9.00 [8.00–11.00]	10.00 [7.00–12.00]	7.00 [6.00–11.00]	7.50 [6.50–9.50]
**Immotility (IM, %)**	27.00 [21.00–35.00]	26.00 [21.00–29.00]	40.00 [26.00–46.00]	28.50 [24.00–44.00]
**Round cells (10^6^ cells/mL)**	0.20 [0.00–1.00]	0.20 [0.10–1.40]	0.20 [0.20–0.40]	1.55 [0.60–3.00] *
**Normal morphology (%)**	4.00 [3.00–4.00]	3.00 [3.00–5.00]	3.00 [2.00–3.00]	2 [1.00–3.00]
**Head defects (%)**	89.00 [87.00–93.00]	89.00 [87.00–93.00]	93.00 [91.00–95.00]	93.00 [92.50–95.50]
**Midpiece defects (%)**	1.00 [0.00–2.50]	1.00 [1.00–1.00]	1.00 [0.00–1.00]	1.00 [0.00–1.50]
**Tail defects (%)**	0.50 [0.00–1.00]	1.00 [0.50–1.00]	0.00 [0.00–1.00]	0.0 [0.00–1.50]

*—significant difference between the COVID−, TUNEL>15 and COVID+, TUNEL>15 groups (*p* ≤ 0.05).

**Table 3:** Median and interquartile range of age, BMI and semen parameters in the groups of men.

Parameters	COVID−, TUNEL<15	COVID+, TUNEL<15	COVID−, TUNEL>15	COVID+, TUNEL>15
**n**	13	9	9	8
**Age (years)**	36.00 [33.00–38.00]	35.00 [34.00–38.00]	34.00 [32.00–37.00]	34.50 [34.00–36.50]
**BMI**	25 [23–25]	23 [23,24]	23 [22–24.5]	24 [23–25]
**Volume (ml)**	3.50 [3.00–4.00]	4.00 [3.00–6.50]	3.00 [2.50–5.00]	3.50 [2.75–3.75]
**Sperm concentration (mln/mL)**	84.00 [67.00–176.00]	98.00 [93.00–146.00]	70.00 [49.00–135.00]	72.00 [59.00–111.50]

## Data Availability

The data that support the findings of this study are available on request from the corresponding author. The data are not publicly available due to privacy or ethical restrictions.
